# Acute angle-closure glaucoma after total knee replacement surgery: case report and literature review

**DOI:** 10.11604/pamj.2022.41.68.31888

**Published:** 2022-01-25

**Authors:** Salma Ketata, Imen Zouche, Rahma Derbel, Rania Dammak, Hichem Kolsi, Ahlem Bousabbeh, Omar Ketata

**Affiliations:** 1 Department of Anesthesia and Critical Care, Habib Bourguiba Hospital, Sfax Tunisia,; 2 Department of Orthopedic Surgery, Habib Bourguiba Hospital, Sfax, Tunisia

**Keywords:** Postoperative, acute angle closure glaucoma, general anesthesia, case report

## Abstract

An early and correct diagnosis improves the prognosis of post-operative Acute angle closure glaucoma (AACG). A 65 years-old monophtalmus man was operated for a total knee replacement surgery, under general anaesthesia without any adverse events. The day after, the patient described recurrent periorbital pain in his eye, with ocular hyperaemia, and reduced visual acuity. A diagnosis of AACG was made and conservative treatment was started to reduce the intraocular pressure. In the post-operative AACG, several predisposing local factors including genetic predisposition, female gender, hypermetropia, increased lens thickness and small corneal diameter, can be added to a pupillary block induced by adrenergic and anticholinergic drugs used in anaesthetic procedures as risk factors. An acute and intensive periorbital or ocular pain, with or without visual disturbance, must aware the physician.

## Introduction

Acute angle closure glaucoma (AACG) after non ocular surgery is a rare complication of general anesthesia. However, in case of delayed diagnosis, it may lead to blindness. Immediate diagnosis and appropriate treatment should be done to prevent visual loss [1]. We present a case of acute angle closure glaucoma after total knee replacement surgery under general anesthesia in a monophtalmus patient. In this case, the most likely trigger was the use of atropine and nefopam.

## Patient and observation

**Patient information**: a 65-year-old monophtalmus man (ASA physical status I, BMI =28,36 kg cm^-2^) with knee arthrosis was scheduled for a total knee replacement surgery under general anesthesia. The preoperative assessment was unremarkable. General anesthesia was induced with IV propofol (3 mg kg^-1^), Fentanyl (3µg kg^-1^), and cisatracurium (0.15 mg kg^-1^) to facilitate tracheal intubation (size 7.5 oral cuffed tracheal tube). Mechanical ventilation was used. Anesthesia was maintained with a mixture of air and oxygen (50%: 50%) supplemented with isoflurane 1 to 1.5 minimum alveolar concentration, Fentanyl and cisatracrium reinjections as needed. Anesthesia lasted almost 3 hours. The patient received 8 mL kg^-1^h^-1^crystalloid infusion during the surgery. The act has occurred with hemodynamic stability. IV paracetamol (1g) and IV nefopam (20 mg) were administered 30 minutes before the end of the surgery and every 6 hours for postoperative pain relief. neostigmine and atropine were injected at the end of surgery for decurarization. No additional drugs were administered.

**Clinical findings**: on the first postoperative day, the patient complained of a reduced visual acuity associated with periorbital pain and nausea.

**Diagnostic assessment**: slit lamp examination revealed lid edema and conjunctival hyperemia. The iris showed fixed and mid-dilated pupil. Gonioscopic examination showed a narrow angle. Intraocular pressure was 30 mm Hg (normal intraocular pressure is 12-20 mm Hg).

**Diagnosis**: the diagnosis of acute angle closure glaucoma was made and in case of delayed diagnosis or absence of treatment, it may lead to blindness.

**Therapeutic interventions**: medical treatment included IV mannitol 20%, tunolol and pilolol eye drops and acetazolamide pills was given to the patient.

**Follow-up**: the next day, the intraocular pressure was normalized, and visual acuity was completely recovered.

**Patient perspective**: the patient did not claim any adverse reaction to the treatment and was satisfied with the result.

**Informed consent**: the patient did finally give his consent.

## Discussion

This case illustrates unilateral AACG most likely related to general anesthesia. The development of AACG requires the coexistence of both a predisposed eye (eye with a narrow anterior chamber angle) and a pupillary block. A pupillary block may appear in different circumstances such as the use of mydriatic agents or a mydriatic situation. Usual risk factors for postoperative AACG are a genetic predisposition, female gender, shallow anterior chamber depth or hypermetropia, increased lens thickness, small corneal diameter, and increased age [2]. Additionally, precipitating factors have been described. There are pharmacologic manipulations of the pupil (Table 1), producing a partially or fully dilated pupil, and emotional factors. These 2 conditions are frequent in the context of general anesthesia [3]. Thirty-six cases of AACG related to anesthesia have been published. The gender distribution is 1 male for 3 females, with a mean age of 63 years (58-64 interquartile range). There were 27 unilateral and 9 bilateral cases. The main identified precipitating factors for the development of AACG were the stress and the use of atropine (80%) or scopolamine. Nine cases (25%) were related to ephedrine use.

**Table 1:** classification of drugs inducing acute angle closure glaucoma by administration route

Route of administration	Type of drug	examples
Eye drops	Mydriatics	Phenylephrine, tropicamide, atropine, homatropine, cyclopentolate
Local drugs	In the anterior chamber	Acetylcholine, carbachol
	Intranasal	Ephedrine,naphazoline, cocaine
	Periocular	Botulinum toxin
	Aerosolized drugs	Salbutamol,albuterol,terbutaline, ipratropium bromide, atropine
Systemic Drugs	Vegetative nerve system drugs	Ephedrine,epinephrine adrenaline
	Anticoagulants	Heparin, warfarin, enoxaparin
	Central nerve system drugs	Topiramate,amphetamines, some antidepressant agents
	Diuretics	Acetazolamide, hydrochlorothiazide
	Other drugs	Cotrimoxazol, histamine H1 and H2 receptor antagonists

In the present case, 3 possible triggering factors which are the stress, the use of atropine for decurarization and nefopam for postoperative analgesia could be incriminated. Nefopam, which is a non-opioid analgesic that inhibits reuptake of serotonin, norepinephrine and dopamine [4], is contraindicated in patients with known angle closure glaucoma because of its parasympatholytic effects. Atropine, which is used to relax the ciliary muscle and dilate the pupil, has long-acting anticholinergic action, and can induce AACG [5]. Moreover, the perioperative period carries the risk of psychological stress. However, general anesthesia and postoperative events often mask the first symptoms. The presence of hypotension or anemia, may enhance ischemic optic neuropathy, which is a much more frequent cause of postoperative vision loss than AACG, but in contrast with AACG, its prognosis is often poor [6,7]. The published cases of AACG are summarized in Table 2 [8-24]. The purpose of this observation is to insist on good preoperative patients´ evaluation. The search for preexisting eye damage would be necessary in a programmed functional surgery. In our case, in front of the existence of monophtalmia, a specialized ophthalmological examination should have been required. In fact, the preoperative diagnosis of glaucoma could change the choice of anesthetic technique and used drugs.

**Table 2:** previously published cases of perioperative acute angle closure glaucoma

Authors (year of publication)	Number of cases (bilateral)	Age, gender, and surgical indication	Identified precipitating factors
Cordier and Vitte (1957) [8]	3 (0)	52-year-old man, inguinal hernia 65-year-old woman, uterine prolapse 54-year-old man, inguinal hernia	“Emotive” patients Atropine sulfate premedication
Gartner and Billet (1958) [9]	4 (1)	54-year-old man, rectal polyp resection 59-year-old woman, cholecystectomy 63-year-old woman, abdominoperineal Resection 60-year-old woman, cholecystectomy	Atropine/scopolamine premedication Pupillary dilatation related to deep anesthesia Anxiety Darkness in the recovery room _ succinylcholine
Wang *et al*. (1961) [10]	5 (0)	60-year-old man, nephrectomy 52-year-old woman, cholecystectomy 72-year-old woman, cholecystectomy 65-year-old man, cystoscopy 69-year-old man, intestinal resection for obstruction	Stress Atropine/scopolamine premedication Succinylcholine
Fazio *et al*. (1985) [11]	9 (2)	7 women and 2 men, mean age 63 year, urologic and gynecologic surgery	7 received atropine or scopolamine, 4 received ephedrine, 6 received succinylcholine
Eldor and Admoni (1989) [12]	2	47-year-old man, closure of ileostomy 64-year-old woman, mastectomy	Atropine premedication, difficult tracheal intubation Reversed with atropine/neostigmine
Lotery and Frazer (1995) [13]	1 (0)	66-year-old woman, abdominal surgery	Atropine premedication Antimuscarinic agents (IV cyclizine and nebulized ipratropium bromide) and nebulized salbutamol
Ujino *et al*. (1997) [14]	1 (0)	49-year-old woman, hip replacement	Atropine premedication High dose of ephedrine
Horimoto *et al*. (1998) [15]	1 (0)	60-year-old woman, surgery for oral Cancer	Atropine premedication Atropine given during general anesthesia
Ates *et al*. (1999) [16]	2(2)	57-year-old woman, cerebral frontal lobe cystic mass resection 54-year-old woman, cholecystectomy	Atropine during induction of anesthesia
Lentschener *et al*. (2002) [17]	1 (0)	66-year-old woman, thyroidectomy	Hypermetropia High dose of ephedrine
Ooi *et al*. (2004) [18]	1 (1)	69-year-old man, coronary artery bypass graft	Use of ephedrine Succinylcholine Reversed with atropine/neostigmine
Ceruti *et al*. (2008) [19]	1(1)	60-year-old woman, brain surgery	Use of ephedrine Associated with subarachnoid hemorrhage
Singer and Salim (2010) [20]	1(1)	68-year- old woman supine surgery	Prone position
Gayat *et al*. (2011) [21]	1(1)	72-year-old woman, cervical spine surgery	Hypermetropia High dose of ephedrine Prone position
LHidalgo Grau *et al*. (2012) [22]	1(0)	90-year-old woman, right hemicolectomy	Stress
Jain *et al*. 2015) [23]	1(0)	9-year-old boy hypospadias surgery	oxybutynin
Stewart *et al*. (2016) [24]	1(0)	65-year-old man lumber supine surgery	Mydriatics agents Prone postioning

## Conclusion

Acute angle closure glaucoma is a rare cause of postoperative visual impairment. The use of mydriatic drugs such as atropine on predisposed individuals may precipitate this acute event, therefore physicians´ awareness is required in order to quickly initiate the treatment.
